# Prognostic value and biological function of LRRN4 in colorectal cancer

**DOI:** 10.1186/s12935-022-02579-x

**Published:** 2022-04-19

**Authors:** Cheng Xu, Yulin Chen, Feiwu Long, Junman Ye, Xue Li, Qiaorong Huang, Dejiao Yao, Xiaoli Wang, Jin Zhao, Wentong Meng, Xianming Mo, Ran Lu, Chuanwen Fan, Tao Zhang

**Affiliations:** 1 College of Medicine, Chengdu University of Traditional Chinese Medicine, Chengdu, 610000 China; 2 Department of Oncology, Hospital of Chengdu University of Traditional Chinese Medicine, Chengdu, 610000 China; 3 Laboratory of Stem Cell Biology, State Key Laboratory of Biotherapy, West China Hospital, Sichuan University, Chengdu, 610000 China; 4 Department of Gastrointestinal, Bariatric and Metabolic Surgery, and Research Center for Nutrition, Metabolism and Food Safety, West China-PUMC CC.C. Chen Institute of Health, West China School of Public Health and West China Fourth Hospital, Sichuan University, Chengdu, 610000 China; 5 Department of Oncology, Affiliated Traditional Chinese Medicine Hospital of Southwest Medical University, Luzhou, 646000 China; 6 Department of Oncology and Department of Biomedical and Clinical Sciences, Linköping University, 58183 Linköping, Sweden; 7 Cancer Center, The General Hospital of Western Theater Command, Chengdu, 610000 China

**Keywords:** Colorectal cancer, Neurogenic biomarker, Leucine-rich repeat neuronal 4, Prognosis, RAS/MAPK signal pathways

## Abstract

**Background:**

Several nervous and nerve-related biomarkers have been detected in colorectal cancer (CRC) and can contribute to the progression of CRC. However, the role of leucine-rich repeat neuronal 4 (LRRN4), a recently identified neurogenic marker, in CRC remains unclear.

**Methods:**

We examined the expression and clinical outcomes of LRRN4 in CRC from TCGA-COREAD mRNA-sequencing datasets and immunohistochemistry in a Chinese cohort. Furthermore, colony formation, flow cytometry, wound healing assays and mouse xenograft models were used to investigate the biological significance of LRRN4 in CRC cell lines with LRRN4 knockdown or overexpression in vitro and in vivo. In addition, weighted coexpression network analysis, DAVID and western blot analysis were used to explore the potential molecular mechanism.

**Results:**

We provide the first evidence that LRRN4 expression, at both the mRNA and protein levels, was remarkably high in CRC compared to controls and positively correlated with the clinical outcome of CRC patients. Specifically, LRRN4 was an independent prognostic factor for progression-free survival and overall survival in CRC patients. Further functional experiments showed that LRRN4 promoted cell proliferation, cell DNA synthesis and cell migration and inhibited apoptosis. Knockdown of LRRN4 can correspondingly decrease these effects in vitro and can significantly suppress the growth of xenografts. Several biological functions and signaling pathways were regulated by LRRN4, including proteoglycans in cancer, glutamatergic synapse, Ras, MAPK and PI3K. LRRN4 knockdown resulted in downregulation of Akt, p-Akt, ERK1/2 and p-ERK1/2, the downstream of the Ras/MAPK signaling pathway, overexpression of LRRN4 leaded to the upregulation of these proteins.

**Conclusions:**

Our results suggest that LRRN4 could be a biological and molecular determinant to stratify CRC patients into distinct risk categories, and mechanistically, this is likely attributable to LRRN4 regulating several malignant phenotypes of neoplastic cells via RAS/MAPK signal pathways.

**Supplementary Information:**

The online version contains supplementary material available at 10.1186/s12935-022-02579-x.

## Background

Tumor progression is a complex and dynamic process of interaction between cancer cells and the tumor microenvironment [1]. Nevertheless, in the last decade, the role of the nervous system, as a crucial component of the tumor microenvironment, has gained much attention in different tumor types, including colorectal cancer (CRC) [2]. The intestine is highly innervated, both from outside the intestines and the enteric nervous system, and increasing evidence has suggested the role of the nervous system as a contributor in CRC [3].

Currently, detecting perineural invasion has been applied to assess risk stratification in CRC patients and is thought to be a sign of tumor metastasis and invasion and a portent of poor prognosis of patients [4, 5]. Although no direct evidence supports the specific bidirectional communication between neurons and cancer cells of CRC, it should exist in theory [6]. Recent studies have indicated that not only can cancer cells stimulate the growth of nerve fibers by secreting neurotrophic factors, but nerve fibers can also infiltrate the tumor microenvironment, stimulating tumor growth and cancer cell dissemination [7]. Meanwhile, several neurogenic biomarkers were significantly associated with the clinicopathological features of CRC patients and even take part in the carcinogenesis and progression of CRC, such as NDRG4, ADRB2, NPY, GABA and et al. [8–11]. In our previous study, we demonstrated that CRC stem cells can generate sympathetic and parasympathetic neurons to comprise the nervous system of CRC tissues. When the expression of the neural marker MAP2 was silenced in human CRC cells, the growth of xenograft tumors was correspondingly inhibited in mouse models, indicating the importance of neurogenic markers in CRC [7]. Although previous studies indicated that the expression of neurogenic molecules in cancer cells is indeed important in CRC, exploring more neurogenic factors is still needed to further understand the crosstalk between cancer cells and neural cells in CRC.

Leucine-rich repeat neuronal 4 (LRRN4), a novel member of the LRRN protein family, was first identified in 2005 [12]. It is expressed in various regions of the central neural system, especially the hippocampus. LRRN4 plays an important role in hippocampus-dependent long-lasting memory [12]. In addition, LRRN4 has been detected in dorsal root ganglion neurons of adult mice and is closely related to the development of dorsal root ganglion [13, 14]. Previous studies also demonstrated that LRRN4 is expressed in various nonneuronal tissues, including the lung, ovary and heart [12, 15]. Apart from the functions of LRRN4 in normal tissue and benign disease, it is also involved in cancers. Recently, a structural alteration of LRRN4 was found in high hyperdiploid acute lymphoblastic leukemia with relapse [16]. LRRN4 has been identified as a marker of primary mesothelial cells, while it was found to be either nondetectable or downregulated in mesothelioma [17]. Although LRRN4 was not highly expressed in normal colon tissue, the level of LRRN4 was much lower in CRC tissue, as detected by the coupling methods of hydroxyapatite chromatography and SDS–PAGE followed by mass spectrometry analysis [18]. However, the clinical significance of LRRN4 remains to be clarified by investigation in a larger sample. Moreover, the functions of LRRN4 are largely unknown.

In this study, we investigated the clinical impact of LRRN4 expression in CRC samples from The Cancer Genome Atlas (TCGA)-COREAD cohort and a Chinese CRC cohort. Then, the effects of LRRN4 on cell proliferation, cell cycle, apoptosis and migration were investigated in CRC cells. Furthermore, a xenograft model was utilized to explore whether LRRN4 impacts xenograft tumor growth in vivo. Finally, we explored the potential molecular mechanism by weighted coexpression network analysis (WGCNA) and the Database for Annotation, Visualization and Integrated Discovery (DAVID). One of the predictive molecular mechanisms was chosen to verify by western blotting analysis after LRRN4 knockdown or overexpression.

## Methods

### Patients

Expression of LRRN4 mRNA and clinicopathological features of TCGA-COREAD patients were obtained from UCSC Xena Browser (https://xenabrowser.net/), and 376 primary CRC tissues and 51 normal mucosal tissues were included. The clinicopathological features included age, gender, overall survival status (OS), progression-free survival status (PFS), microsatellite instability (MSI) status, tumor location, histological type, pathologic stage, lymphatic invasion, perineural invasion, venous invasion and adjuvant chemoradiotherapy.

Expression of LRRN4 protein and clinicopathological features of 81 primary CRC tissues and corresponding distant normal mucosa tissues were obtained from West China Hospital, Sichuan University. Informed consent was obtained from all patients. The protocol conformed to the Declaration of Helsinki, and all tests were approved by the Institutional Review Board of West China Hospital, Sichuan University. The clinicopathological features were obtained from patient medical records, including age, gender, tumor location, histological type, pathologic stage, grade, and adjuvant chemoradiotherapy. Patients were followed up to obtain information on OS and PFS status.

### Immunohistochemistry

Immunostaining analysis of LRRN4 was performed as described in our previous study [19]. The primary anti-LRRN4 antibody (Abcam, UK, ab133372) was used at a 1:200 dilution and the DAB kit (GeneTech, China) was used following the protocol. All slides were scored 0–4 according to the intensity by two independent investigators, 0 represented the weakest intensity, 4 represented the strongest intensity. X-tile was used to generate the optimal cutoff score with low LRRN4 expression (0–2 score) and high LRRN4 expression (3–4 score).

### Cell culture and transduction

The CRC cell line Caco2 (SCSP-5027) was obtained from Shanghai Institute for Biological Sciences, Chinese Academy of Science. SW480 (CCL-228™), HCT-116 (CCL-247™), LoVo (CCL-229™) and HIEC-6 (CRL-3266™) cells were obtained from the American Type Culture Collection. These cells were grown in Dulbecco's modified Eagle's medium (DMEM; HyClone, USA) supplemented with 10% fetal bovine serum (GEMINI, USA) and a 1% penicillin–streptomycin mixture (HyClone, USA). All cells were cultured at 37 °C in a humidified atmosphere of air containing 5% CO_2_. STR profiling and mycoplasma contamination were performed to maintain the authenticity of cell line on a regular basis.

Knockdown and overexpression of LRRN4 were achieved by lentiviral transduction. The sequence of LRRN4 was obtained from the National Center for Biotechnology Information. Lentiviruses containing LRRN4 small hairpin RNA (shRNA) for knockdown were constructed by GeneChem (China). The plasmid for overexpression of LRRN4 was constructed by Sangon Biotech (China), and lentiviruses for overexpression of LRRN4 were produced and titered as described elsewhere[7].

### RNA extraction and real-time quantitative PCR

Total RNA of CRC cells was extracted with TRIzol reagent (Molecular Research Center, USA) according to the manufacturer’s instructions. After confirming the RNA quality, cDNA was synthesized using the PrimeScript RT Reagent Kit (Takara Bio, Japan). Gene expression differences were detected by real-time quantitative PCR using SYBR Green Master Mixture (Roche, Switzerland). The primers were 5ʹ-CTTGCTTCTGTCGCCACACAC-3ʹ (forward) and 5ʹ-AGGAGCCAAGACAAGTCACA-3ʹ (reverse). The data were normalized to the expression of the housekeeping gene GAPDH. The relative gene expression levels were calculated using the 2^−∆∆Ct^ method.

### Western blot analysis

Equal amounts of total protein were separated by SDS–PAGE and electrophoretically transferred to polyvinylidene difluoride membranes (Millipore, USA). The membranes were blocked with 5% albumin from bovine serum and then incubated overnight at 4 °C with the indicated primary antibodies, LRRN4 (Abcam, UK, ab133372), proliferating cell nuclear antigen (PCNA) (Calbiochem, Germany, 07-2162),CHK1 (Beyotime Biotechnology, China, AF18849), Phospho-Chk1 (p-Chk1, Cell Signaling Technology, USA, 2341S), Bcl-2 (Cell Signaling Technology, USA, 3498S), Bcl-xl (Abmart, China, Q07817), Caspase 3(Caspase 3/p17/p19 Monoclonal antibody, Proteintech, USA, 66470-2-Ig), Cleaved Caspase-3 (Cell Signaling Technology, USA, 9961S), Akt (Cell Signaling Technology, USA, 4691S), Phospho-Akt (p-Akt, Cell Signaling Technology, USA, 9271S), ERK 1/2 (Santa Cruz, UK, SC-514302), p-ERK 1/2(Santa Cruz, UK, SC-81492). After the appropriate secondary antibodies were added at room temperature, the proteins were detected with ECL reagent (SuperSignal West Pico Chemiluminescent Substrate, Pierce Biotechnology, USA) and visualized with the Electrophoresis Gel Imaging Analysis System (DNR Bio-Imaging Systems, Neve Yamin, Israel).

### Colony formation assay

For the colony formation assay, cells (500 cells per well) were incubated in 6-well plates and allowed to grow until the colony can be recognized. Cells were washed with PBS three times and fixed using 4% paraformaldehyde for 30 min followed by staining with crystal violet at room temperature for 30 min. The plates were then rinsed with distilled water and dried before the count. Colonies containing more than 50 cells in each well were counted.

### Flow cytometry

EdU (5-ethynyl-20-deoxyuridine) incorporation assay was performed using an EdU assay kit (YF®647A Click-iT EdU Imaging Kits; US Everbright, China) following the manufacturer's guidelines. Briefly, cells were incubated with 10 µM EdU and subsequently fixed in 4% paraformaldehyde. After EdU staining, cell nuclei were stained with DAPI, and cell proliferation was detected by a BD FACS Canto™ System (BD Biosciences, USA).

DNA content analysis was performed using a cell cycle analysis kit (Sangon Biotech, China) following the manufacturer's guidelines. Briefly, cells were dissociated by trypsin and fixed with chilled 70% ethanol overnight. The staining working fluid of propidium iodide (PI) and RNase A was used to stain DNA for 30 min. Cells were washed and filtered through a 40 µm cell strainer before flow cytometry. Cell cycle distributions were then analyzed by a BD Accuri™ C6 Plus flow cytometer (BD Biosciences, USA).

Cell apoptosis was detected using YF®647A-Annexin V and PI Apoptosis Kit (US Everbright, China) following the manufacturer’s instructions except that the cell nuclei staining dye was changed from PI (supplied with the kit) to DAPI (Beyotime, China). After staining, the activity of Annexin V/DAPI was then examined using a BD FACS Canto™ System (BD Biosciences, USA).

### Wound healing assay

Cells were seeded into six-well plates for adherent culture. When cells reached 80% confluence, 200 µl pipette tips were used to make a thin wound. Then, the detached cells were washed off twice and incubated with basic DMEM. After that, images were acquired at 0, 24 and 48 h after wounding using a phase-contrast microscope. The relative wound healing closure was calculated by measuring the area of the gap at 0, 24 and 48 h.

### Xenograft model

Male severe combined immune deficiency (SCID) mice, 4–6 weeks old, were purchased from Beijing Vital River Laboratory Animal Technology (China) and housed under pathogen-free conditions. Animal studies were approved by the Institutional Review Board of West China Hospital, Sichuan University. Caco2 cells (3 × 106) were suspended in PBS and mixed with Matrigel (Corning, USA) at a 5:2 ratio. Then, the cells were injected subcutaneously into the right flank of each mouse. The large diameter and small diameter of tumors were monitored twice a week. The tumor volumes were calculated by the formula (π)/6 × (large diameter) × (small diameter)2. When the established criteria for the endpoint were reached, mice were anesthetized according to the 2020 AVMA Guidelines on Euthanasia state. In short, the mice were anesthetized by intraperitoneal injection of 0.1 mL of 1% phenobarbital sodium, and then the tumors were dissected and weighed. The paraffin-embedded sections were prepared and used for histological and immunohistochemistry analysis. The slices were cut into 5 μm sections and stained with hematoxylin–eosin for histological evaluation.

### WGCNA and DAVID

The R package WGNCA was used for the enrichment of the coexpression network of LRRN4 as previously described[20]. In short, genes with log2 (RSEM > 1) from TCGA-COREAD were included in the network. A power of 3 was selected to compute into a topological overlap matrix, with over 50 genes for a dendrogram, using the function topological overlap matrix similarity. Similar modules were merged at a cutoff (< 0.25) to obtain moderately large and distinct modules. Then, the correlations among gene expression modules and clinical traits were calculated using the module-trait relationships of WGCNA. The tumor location, MSI status, histological type, pathological stage, lymphatic invasion, perineural invasion, venous invasion, and preoperative CEA were chosen as clinical traits. In addition, the association of gene significance (GS) and module membership (MM) was also assessed to determine how close the significance of gene expression is to the magenta module. Finally, to further explore the biofunction of LRRN4 by bioinformatics, Gene Ontology (GO) enrichment and Kyoto Encyclopedia of Genes and Genomes (KEGG) pathway analyses were employed using the DAVID database. GO annotation included cellular component (CC), biological process (BP) and molecular function (MF) terms. A *p* value cutoff of 0.05 was used for significant enrichment.

### Statistical analysis

All statistical analyses were performed using R statistical software (version 4.0.5), SPSS software (version 27.0) and GraphPad Prism software (version 7.00). LRRN4 expression values were RSEM-normalized and shown as log2 values, analyzed in UCSC Xena Browser. The χ^2^ test method was used to determine the difference in the expression of LRRN4 among normal mucosa and tumors, as well as the relationship of LRRN4 expression in CRC with clinicopathological features. Kaplan–Meier analyses and log-rank tests were used to estimate the survival curves. Univariate and multivariate analyses were employed to establish a Cox proportional hazard regression model alone or after adjusting for clinical variables. In the analyses of results obtained from experiments in cells, the data are presented as the mean ± SD of at least three independent assays. Statistical comparisons between groups were performed using the unpaired Student’s t test or the Mann–Whitney U test. A *p* value less than 0.05 was considered statistically significant.

## Results

### LRRN4 is highly expressed in CRC and correlates with some clinicopathological features

**Fig. 1:**
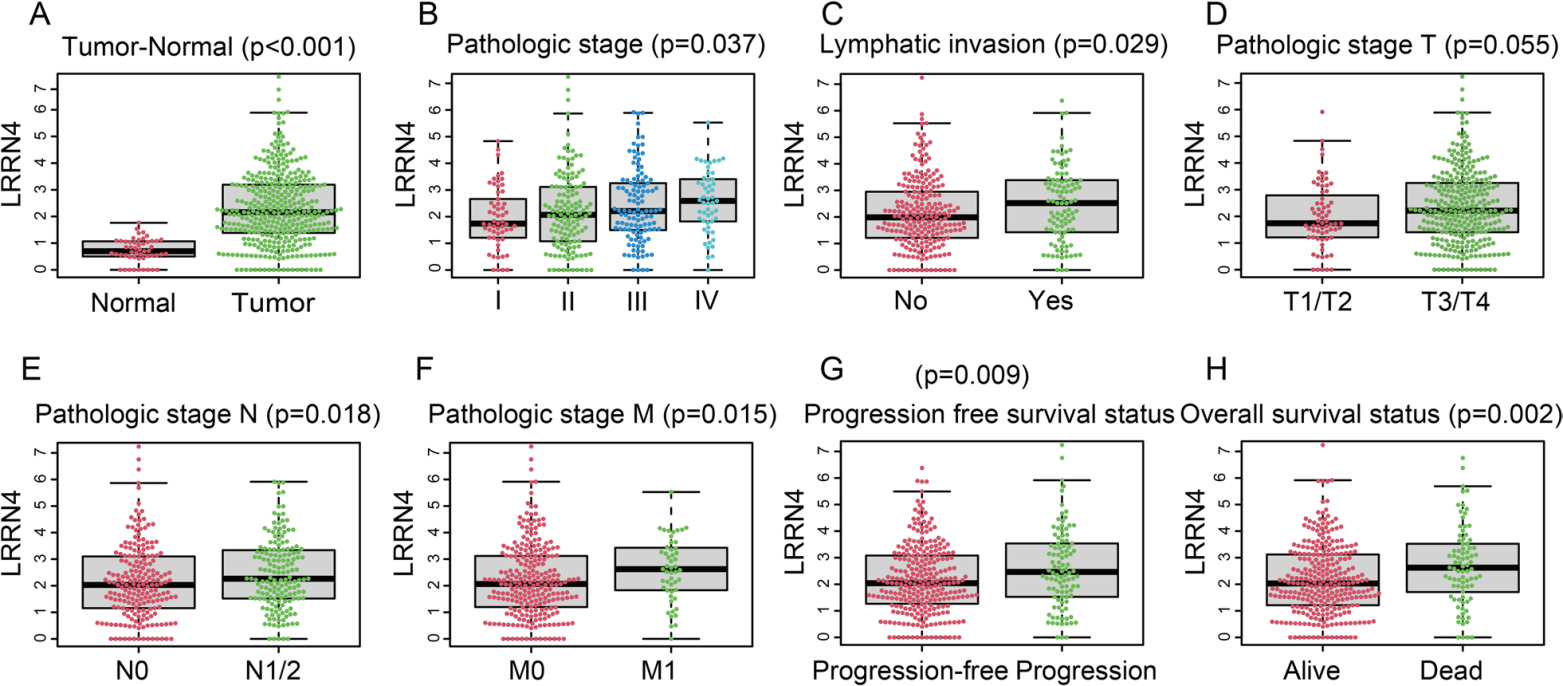
Expression of LRRN4 in CRC tissue with different clinicopathological characteristics from the TCGA-COREAD cohort. **A** Expression of LRRN4 in CRC and normal colorectal tissues. LRRN4 expression in CRC tissue of different pathological stages (**B**), lymphatic invasion (**C**), pathological T stage (**D**), pathological N stage (**E**), pathological M stage (**F**), progression-free survival status (PFS) (**G**) and overall survival status (OS) (**H**)

To investigate the potential significance of LRRN4 in CRC patients, the relationship between LRRN4 expression and clinicopathological characteristics was analyzed. As shown in Fig. 1, the expression level of LRRN4 was much higher in CRC than in normal colorectal tissue (p < 0.001). Moreover, LRRN4 expression was significantly different in different pathological stages (p = 0.037), lymphatic invasion (p = 0.029), progression-free survival status (PFS) (p = 0.009), and overall survival status(OS)(p = 0.002). No significant difference was observed in the rest of the clinicopathological parameters, including age, gender, location, microsatellite instability status (MSI), histological type, perineural invasion and venous invasion (Additional file 1: Fig. S1).

### High LRRN4 mRNA expression is an independent prognostic factor for poor PFS and OS in CRC patients

**Fig. 2:**
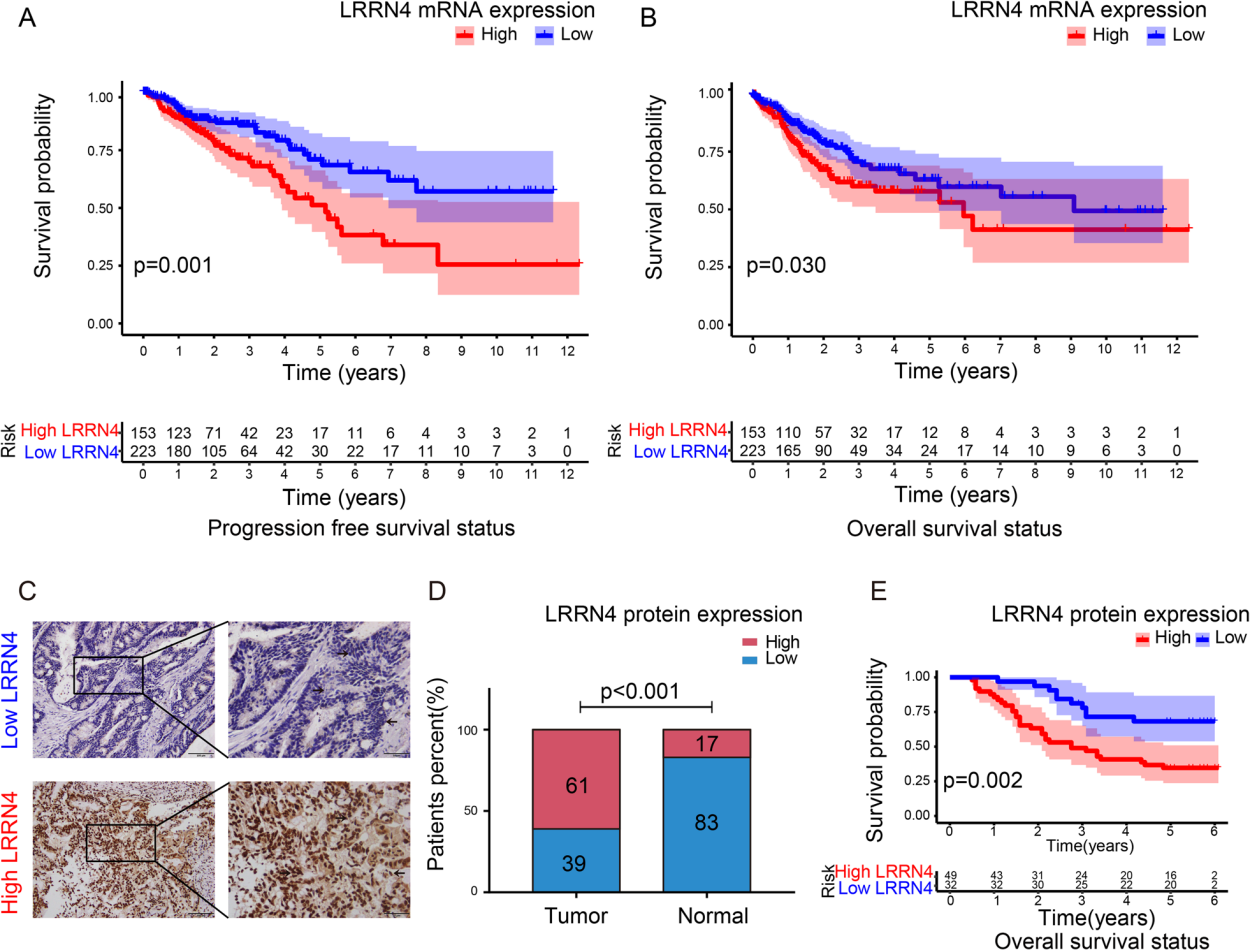
LRRN4 expression positively correlated with the clinical outcome of CRC patients at both the mRNA and protein levels. **A** Kaplan–Meier curves of PFS of CRC from TCGA based on the expression of LRRN4 mRNA, **B** Kaplan–Meier curves of OS of CRC from TCGA based on the expression of LRRN4 mRNA, **C** Representative IHC images with low expression and high expression of LRRN4 protein. Magnification 200 × , scale bars correspond to 100 μm (left) and magnification 400 × , scale bars correspond to 50 μm (right). Arrows indicate cells with low or high expression of LRRN4. **D** Patients percent with high or low LRRN4 expression in CRC and normal colorectal tissues. **E** Kaplan–Meier curves of OS of CRC in a Chinese cohort based on the expression of LRRN4 protein

To investigate the potential survival significance of LRRN4 expression, we performed Kaplan–Meier survival analysis. The patients were first classified into low- and high-LRRN4 expression groups using X-tile plots to generate the optimal cutoff score. The survival analysis showed that high LRRN4 expression was associated with poor PFS (Fig. 2A,  *p* = 0.001) and OS (Fig. 2B,  *p* = 0.030).

To further assess whether LRRN4 expression could independently predict PFS and OS in CRC patients, both univariate and multivariate Cox regression analyses were performed by adjusting for gender, age, stage, MSI status, lymphatic invasion, venous invasion and adjuvant chemoradiotherapy as covariates. Univariate analyses showed that high LRRN4 expression was significantly associated with poor PFS (Table 1, *p* = 0.015) and OS (Table 2, *p* = 0.005). In multivariate analyses, high expression of LRRN4 remained a strong prognostic value for PFS (Table 1, HR = 1.797,95% CI = 1.009–3.200, *p* = 0.047) and OS (Table 2, HR = 1.733, 95% CI = 1.014–2.961, *p* = 0.044), even after adjusting for other covariates, indicating its potential prognostic value for PFS and OS in CRC patients.

**Table 1:** Univariate and multivariate Cox proportional hazards regression analyses for PFS of LRRN4 and clinical features

Variables	Univariable Cox			Multivariable Cox		
	HR	95%CI	*p* value	HR	95%CI	*p* value
LRRN4 (high vs. low)	1.992	1.143–3.469	**0.015**	1.797	1.009–3.200	**0.047**
MSI (high vs. low)	0.677	0.316–1.448	0.314	1.209	0.524–2.792	0.656
Age	1.346	0.753–2.407	0.316	1.020	0.997–1.044	0.092
Gender (male vs. female)	1.348	0.772–2.354	0.294	1.118	0.619–2.020	0.712
Histological type (nonmucinous vs. mucinous)	0.394	0.121–1.278	0.121	0.425	0.128–1.410	0.162
Pathologic stage (III/IV *vs.* I/II)	2.900	1.620–5.192	**0.000**	1.707	0.814–3.578	0.157
Lymphatic invasion (yes vs. no)	2.348	1.346–4.095	**0.003**	1.067	0.423–2.690	0.890
Perineural invasion (yes vs. no)	1.932	1.093–3.418	**0.024**	1.268	0.637–2.522	0.499
Venous invasion (yes vs. no)	2.045	1.170–3.575	**0.012**	1.215	0.523–2.824	0.650
Adjuvant chemoradiotherapy (no vs. yes)	3.540	1.184–6.652	**0.000**	2.781	1.289–6.000	**0.009**

A *p* value below 0.05 was considered significant and highlighted (bold)

*HR* hazard ratio, *CI* confidence interval

**Table 2:** Univariate and multivariate Cox proportional hazards regression analyses for OS of LRRN4 and clinical features

Variables	Univariable Cox			Multivariable Cox		
	HR	95%CI	*p* value	HR	95%CI	*p* value
LRRN4 (high vs. low)	2.063	1.247–3.413	**0.005**	1.733	1.014–2.961	**0.044**
MSI (high vs. low)	0.913	0.463–1.801	0.792	0.957	0.449–2.039	0.909
Age	1.032	1.011–1.053	**0.002**	1.035	1.012–1.059	**0.003**
Gender (male vs. female)	1.667	0.993–2.798	0.053	1.541	0.898–2.644	0.116
Histological type (mucinous vs. nonmucinous)	1.239	0.588–2.611	0.573	1.254	0.570–2.760	0.574
Pathologic stage (III/IV *vs.* I/II)	2.914	1.723–4.927	**0.000**	4.691	2.333–9.431	**0.000**
Lymphatic invasion (yes vs. no)	2.025	1.214–3.377	**0.007**	0.633	0.268–1.496	0.298
Veno ius invasion (no vs. yes)	0.990	0.597–1.640	0.968	2.180	0.949–5.005	0.066
Adjuvant chemoradiotherapy (no vs. yes)	2.481	1.487–4.138	**0.001**	0.540	0.291–1.001	0.051

A *p* value below 0.05 was considered significant and highlighted (bold)

*HR* hazard ratio, *CI* confidence interval

### The prognostic significance of LRRN4 for CRC patients was validated at the protein level

Having found the significance of the expression of LRRN4 mRNA in CRC prognosis, we further investigated the expression of LRRN4 protein in samples from 81 CRC patients by immunohistochemistry (Fig. 2C). We first analyzed the expression levels of LRRN4 in CRC and normal mucosa tissues. Consistent with the results of the TCGA data analysis, there were more patients (83%) with high LRRN4 expression in CRC than in normal colorectal tissue (39%) (Fig. 2D,  p < 0.001). Then, the correlation of LRRN4 expression and clinical characteristics in CRC patients was analyzed. The results showed that high LRRN4 expression was statistically related to pathological stage (Table 3, *p* = 0.005), lymph node metastasis (Table 3, *p* = 0.006) and OS status (Table 3, *p* = 0.003). Next, we performed survival analysis and found that high LRRN4 expression was correlated with poor PFS (Additional file 2: Fig. S2, *p* = 0.064) and OS (Fig. 2E, * p* = 0.002), although no statistical significance was found in PFS. Then, a Cox proportional hazards model was used to analyze the impact of various clinical and pathological parameters on patient survival. Univariate Cox regression analyses showed that LRRN4 (Table 4, HR = 2.936, 95% CI = 1.441–5.982, *p* = 0.003) and Pathologic stage (Table 4, HR = 3.189, 95% CI = 1.564–6.501, *p* = 0.001) that were risk factors for poor OS. Furthermore, multivariate Cox regression analyses showed that LRRN4 expression (HR = 2.364, 95% CI = 1.104–5.062, *p* = 0.027) was independent risk factor for poor OS (Table 4) by adjusting for gender, age, location, histological type, pathologic stage, grade and adjuvant chemoradiotherapy as covariates, indicating the significance of LRRN4 expression for outcomes in CRC patients.

**Table 3:** The correlation of LRRN4 expression and clinical characteristics in crc patients

Characteristics	LRRN4-Low (%)	LRRN4-High (%)	*p* value
Gender			
Male	17 (53.1)	28 (57.1)	0.820
Female	15 (46.9)	21 (42.9)	
Age			
≤ 65	19 (59.4)	31 (63.3)	0.816
> 65	13 (40.6)	18 (36.7)	
Overall survival status			
Alive	22 (68.8)	17 (34.7)	**0.003**
Death	10 (31.2)	32 (65.3)	
Progression-free survival status			
Progression-free	20 (64.5)	13 (39.4)	0.051
Progression	11 (35.5)	20 (60.6)	
Location			
Colon	20 (62.5)	37 (75.5)	0.225
Rectum	12 (37.5)	12 (24.5)	
Histological type			
Mucinous	9 (29.0)	17 (32.5)	0.633
Nonmucinous	22 (71.0)	32 (67.5)	
Pathologic stage-T			
T 1/2	6 (18.8)	5 (10.2)	0.328
T 3/4	26 (81.3)	44 (89.8)	
Pathologic stage-N			
N0	21 (65.6)	16 (32.7)	**0.006**
N1/2	11 (34.4)	33 (67.3)	
Pathologic stage-M			
M0	31 (96.9)	41 (83.7)	0.080
M1	1 (3.1)	8 (16.3)	
Pathologic stage			
Stage I	5 (15.6)	3 (6.1)	**0.005**
Stage II	16 (50.0)	10 (20.4)	
Stage III	10 (31.3)	28 (57.2)	
Stage IV	1 (3.1)	8 (16.3)	
Adjuvant chemoradiotherapy			
No	8 (25.0)	19 (38.8)	0.234
Yes	24 (75.0)	30 (61.2)	
Grade			
1/2	29 (93.5)	42 (89.4)	0.697
3	2 (6.5)	5 (10.6)	

A *p* value below 0.05 was considered significant and highlighted (bold)

*HR* hazard ratio, *CI* confidence interval

**Table 4:** Univariate and multivariate Cox proportional hazards regression analyses for OS of LRRN4 and clinical features

Variables	Univariable Cox			Multivariable Cox		
	HR	95%CI	p value	HR	95%CI	p value
LRRN4 (high vs. low)	2.936	1.441–5.982	**0.003**	2.364	1.104–5.062	**0.027**
Age	1.004	0.983–1.026	0.713	1.003	0.983–1.024	0.769
Gender (female vs. male)	0.766	0.413–1.418	0.396	0.891	0.462–1.720	0.731
Location (rectum vs. colon)	1.049	0.545–2.019	0.885	1.400	0.692–2.829	0.349
Histological type (nonmucinous vs. mucinous)	1.077	0.566–2.046	0.822	0.914	0.425–1.965	0.818
Pathologic stage (III/IV vs. I/II)	3.189	1.564–6.501	**0.001**	2.282	1.051–4.952	**0.037**
Adjuvant chemoradiotherapy (yes vs. no)	0.721	0.387–1.345	0.304	0.748	0.385–1.454	0.392
Grade (1/2 vs. 3)	0.424	0.102–1.758	0.237	0.406	0.090–1.823	0.239

p value below 0.05 was considered significant and highlighted (bold)

*HR* hazard ratio, *CI* confidence interval

### LRRN4 is highly expressed in CRC cell lines and LRRN4 promotes cell proliferation

**Fig. 3:**
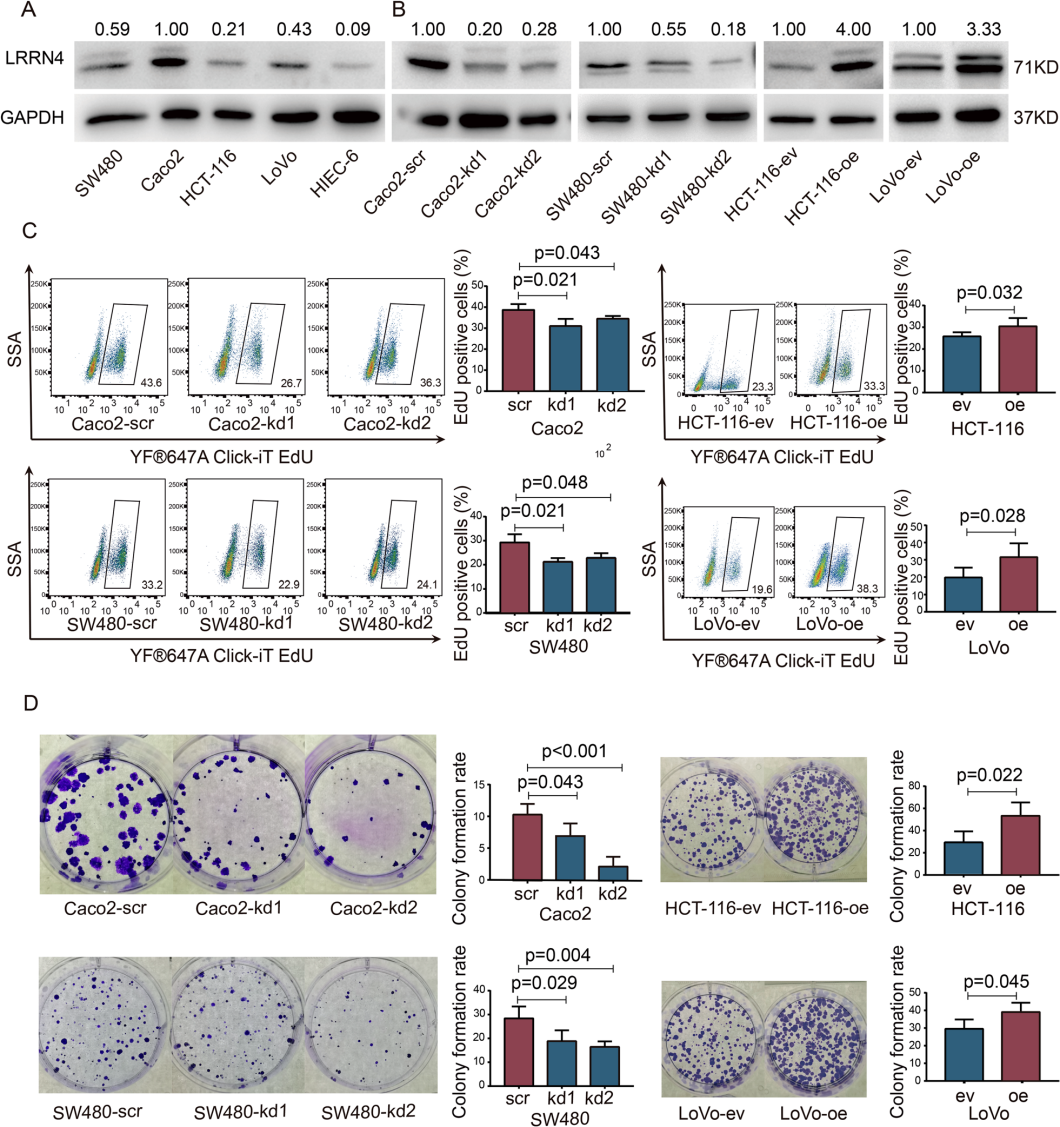
LRRN4 is highly expressed in CRC cell lines and LRRN4 promotes cell proliferation. **A** Expression of LRRN4 in SW480, Caco2, HCT-116, LoVo and HIEC-6 cells. **B** LRRN4 expression in Caco2 and SW480 cells with constitutive expression of two different LRRN4 shRNAs (kd1 or kd2), and LRRN4 expression in HCT-116 and LoVo cells constitutively expressing LRRN4 open reading frame (oe). **C** EdU proliferation assay and quantification in Caco2 and SW480 cells with LRRN4 knockdown and in HCT-116 and LoVo cells overexpressing LRRN4. **D** Colony formation assay of cell clonal proliferation ability in Caco2 and SW480 cells with knockdown of LRRN4 and in HCT-116 and LoVo cells overexpressing LRRN4. Bars represent the means with SEM from at least three independent experiments

In light of our above results in CRC patients suggesting that LRRN4 was closely correlated with the stage and prognosis of CRC, we further conducted cell experiments to explore the potential biological function of LRRN4 in CRC cells. We initially investigated LRRN4 expression in CRC cell lines (Caco2, SW480, HCT-116, and LoVo) and normal colorectal epithelial cells (HIEC-6) (Fig. 3A). The expression of LRRN4 was higher in CRC cells than in HIEC-6 cells. In CRC cell lines, LRRN4 was highly expressed in Caco2 and SW480 cells compared to HCT-116 and LoVo cells. Therefore, we generated sublines of Caco2- and SW480-silenced LRRN4 cells, which were infected with lentivirus expressing two different LRRN4-shRNAs (Caco2-kd1 and Caco2-kd2; SW480-kd1 and SW480-kd2) and the corresponding control (Caco2-scr, SW480-scr). Meanwhile, HCT-116 and LoVo cells were infected with lentiviruses overexpressing LRRN4 (HCT-116-oe and LoVo-oe) and the corresponding controls (HCT-116-eV and LoVo-ev), respectively. The knockdown of LRRN4 in Caco2 and SW480 cells and overexpression of LRRN4 in HCT-116 and LoVo cells were validated by real-time quantitative PCR (Additional file 3: Fig. S3) and Western blot (Fig. 3B).

To determine the role of LRRN4 in cell proliferation, we carried out an EdU assay. As shown in Fig. 3C, knockdown of LRRN4 significantly suppressed the proliferation of Caco2 and SW480 cells (Caco2-kd1, Caco2-kd2, SW480-kd1 and SW480-kd2 vs. Caco2-scr, SW480-scr), while LRRN4 overexpression promoted the proliferation of HCT-116 and LoVo cells (HCT-116-oe, LoVo-oe vs. HCT-116-v, LoVo-ev). Moreover, we investigated the capacity of colony formation in cell lines with different LRRN4 expression. The results showed that knockdown of LRNN4 significantly decreased the colony formation rate in CRC cells (Caco2-kd1, Caco2-kd2, SW480-kd1 and SW480-kd2 cells vs. Caco2-scr, SW480-scr) (Fig. 3D). However, an increase in the number of colonies was observed in HCT-116 and LoVo cells with LRRN4 overexpression compared to corresponding control cells (HCT-116-eV and LoVo-ev) (Fig. 3D), which further verified the promotive effect of LRRN4 on cell proliferation.

### LRRN4 promotes cell DNA synthesis and inhibits apoptosis in CRC cells

**Fig. 4:**
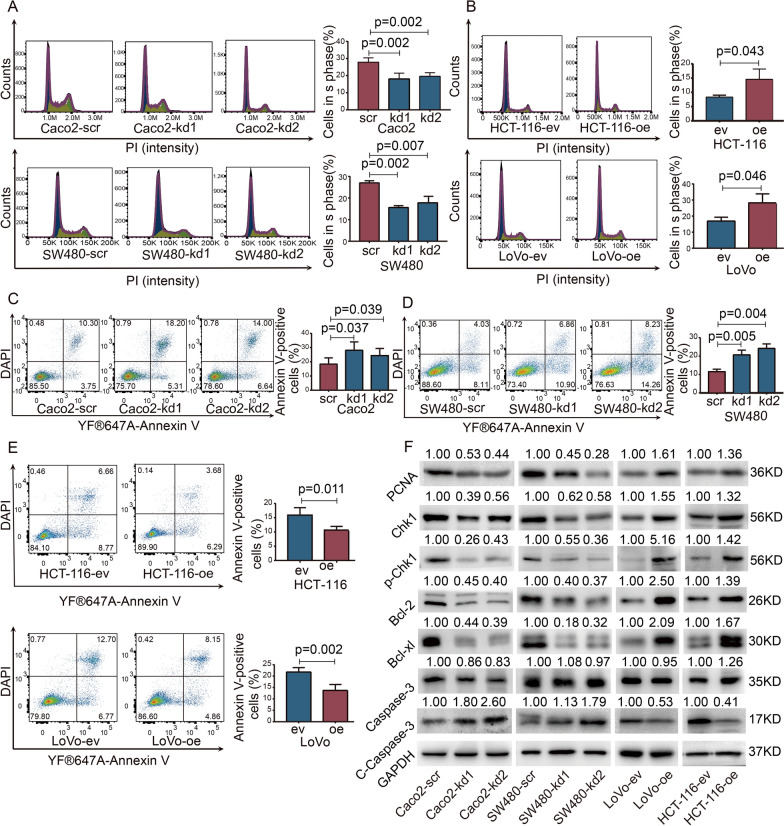
LRRN4 promotes cell DNA synthesis and inhibits apoptosis in CRC cells. **A** Representative histogram of the gated cells in the G0/G1, S and G2/M phases and quantitative analysis of the S phase proportion in Caco2 and SW480 cells with LRRN4 knockdown. **B** Representative histogram of the gated cells in the G0/G1, S and G2/M phases and quantitative analysis of the S phase proportion in HCT-116 and LoVo cells overexpressing LRRN4. **C** Representative plots of YF®647A-Annexin V flow cytometry and DAPI staining experiments and quantitative analysis of Annexin V-positive Caco2 with LRRN4 knockdown. **D** Representative plots of YF®647A-Annexin V flow cytometry and DAPI staining experiments and quantitative analysis of Annexin V-positive SW480 with LRRN4 knockdown. **E** Representative plots of YF®647A-Annexin V flow cytometry and DAPI staining experiments and quantitative analysis of Annexin V-positive HCT-116 and LoVo cells overexpressing LRRN4. **F** Representative images of Western blot analyses of PCNA, Chk1, p-Chk1, Bcl-2, Bcl-xl, caspase 3 and cleaved caspase 3 (c-caspase3) in CRC cells with knockdown or overexpression of LRRN4. Bars represent the means with SEM from at least three independent experiments

The role of LRRN4 in CRC cell proliferation could be regulating the cell cycle or cell apoptosis. Therefore, the cell cycle and apoptosis were analyzed using flow cytometry. The cell cycle distribution analysis showed significantly decreased cell populations in the S phase in both Caco2 and SW480 cells with knockdown of LRRN4 (Caco2-kd1, Caco2-kd2, SW480-kd1 and SW480-kd2) compared to their corresponding control cells (Caco2-scr, SW480-scr) (Fig. 4A). In contrast, overexpression of LRRN4 induced a higher percentage of S phase distribution in HCT-116 and LoVo cells than in their controls (Fig. 4B). The quantitative analyses of cells in GO/G1 and G2/M phases were showed in Additional file 1: Fig. S4. We next analyzed whether LRRN4 exerted any impacts on cell apoptosis. Compared to their corresponding controls, knockdown of LRNN4 resulted in a higher percentage of apoptotic Caco2 and SW480 cells (Fig. 4C, D). In contrast, the rates of apoptotic cells were significantly decreased in CRC cells overexpressing LRRN4 (HCT-116-oe and LoVo-oe) compared to control cells (HCT-116-eV and LoVo-ev) (Fig. 4E). Taken together, these data indicated that LRRN4 plays a functional role in promoting DNA synthesis and inhibiting cell apoptosis.

**Fig. 5:**
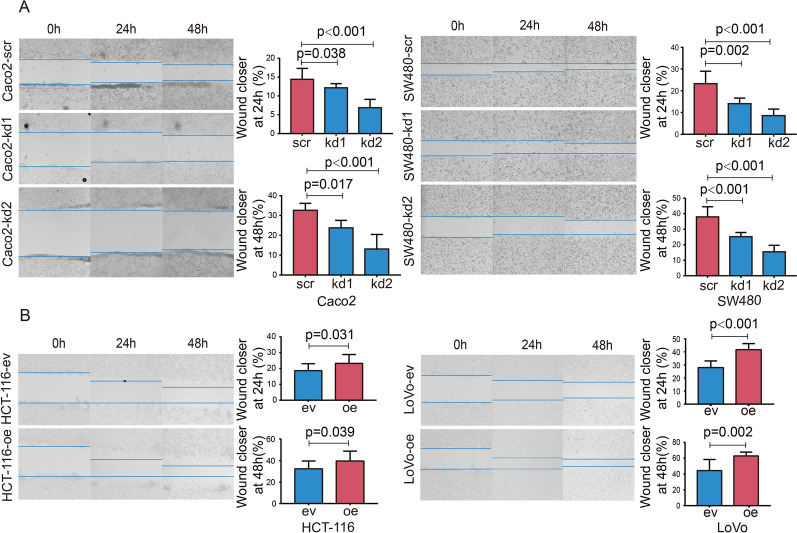
LRRN4 accelerates cell migration. **A** Representative images of scratched monolayer re-epithelialization and quantitative analysis in Caco_2_ and SW480 cells with knockdown of LRRN4. **B** Representative images of scratched monolayer re-epithelialization and quantitative analysis in HCT-116 and LoVo cells overexpressing LRRN4. Bars represent the means with SEM from at least three independent experiments

To further verify whether LRRN4 is involved in these biological processes, western blot analyses were performed to check the related protein. Knocking down LRRN4 inhibited the expression of PCNA, Chk1 and p-Chk1, while overexpression of LRRN4 led to the opposite results, indicating the role of LRRN4 in DNA synthesis and cell cycle. Moreover, knocking down LRRN4 inhibited the expression of Bcl-2, Bcl-xl and upregulated the expression of cleaved-caspase3 (c-caspase3) (Staurosporine has been used as the positive control and the results were shown in Additional file 5: Fig. S5), validating the role of LRRN4 in apoptosis (Fig. 4F).

### LRRN4 accelerates cell migration

To further explain the correlation between LRRN4 and pathological stage in CRC patients at the cellular level, a wound healing assay was employed to evaluate the effect of LRRN4 on cell migration. Knockdown of LRRN4 significantly impaired the migratory capacity of Caco2 and SW480 cells, resulting in impaired wound closure at two different time points (24 h and 48 h) (Fig. 5A). To confirm the above findings, cell migration was determined in CRC cells overexpressing LRNN4. The results showed that overexpression of LRRN4 significantly accelerated wound closure compared to the control, indicating a promotive effect of LRRN4 on cell migration in HCT-116 and LoVo cells (Fig. 5B).

### LRRN4 accelerates xenograft tumor growth in vivo

**Fig. 6:**
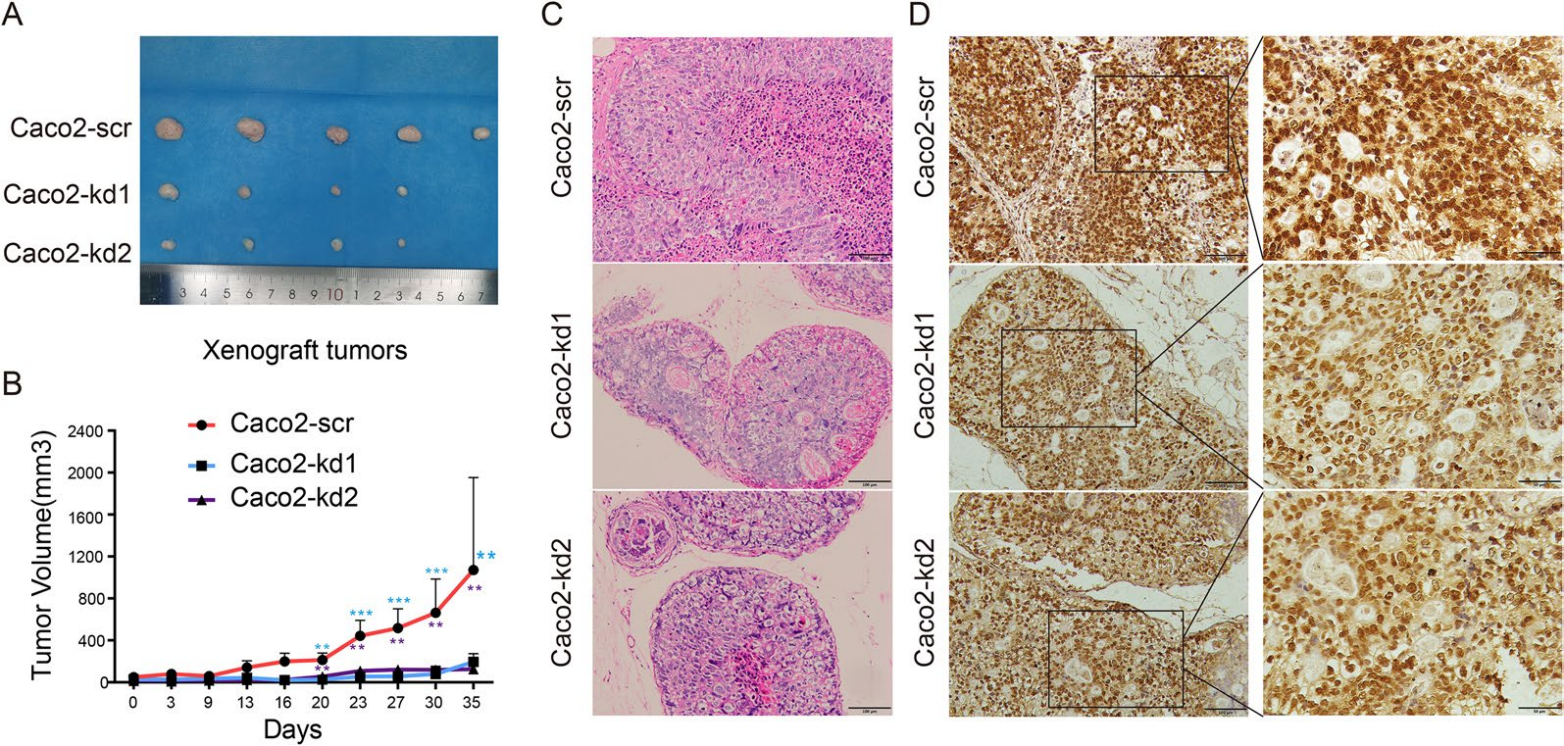
Effects of LRRN4 on Caco2 xenograft tumor growth in SCID mice. **A** Image of harvested xenograft tumors from SCID mice in each group (Caco2-scr, Caco2-kd1 and Caco2-kd2) at necropsy. **B** Tumor volume (mm^3^) of xenografts with Caco2-scr, Caco-kd1 and Caco2-kd2 as measured twice a week. **C** The histology images of xenograft tumors. Magnification 200 × , scale bars correspond to 100 μm. D Expression of LRRN4 in xenograft tumors (Caco2-scr, Caco2-kd1 and Caco2-kd2). Magnification 200 × , scale bars correspond to 100 μm (left) and magnification 400 × , scale bars correspond to 50 μm (right). *, *p* < 0.05; **, *p* < 0.01; ***, *p* < 0.001

After demonstrating the positive impact of LRRN4 on CRC cells in vitro, we sought to confirm its role in a more physiologically relevant in vivo model. A xenograft model was utilized to investigate whether LRRN4 impacts tumor growth in vivo. Cells were injected subcutaneously into SCID mice. As shown in Fig. 6A, the sizes of xenograft tumors of cells with knockdown of LRRN4 (Caco-kd1 and Caco2-kd2) were much smaller than that of the control (Caco2-scr). In the growth curve analyses (Fig. 6B), tumor growth was significantly inhibited by knockdown of LRRN4, indicating the promoting role of LRRN4 in CRC xenograft tumors. The histology images of xenograft tumors were showed in Fig. 6C and the Fig. 6D showed the maintenance of LRRN4 knockdown in tumors of Caco-kd1 and Caco2-kd2.

### LRRN4 affects several cancer-related biological functions and pathways

**Fig. 7:**
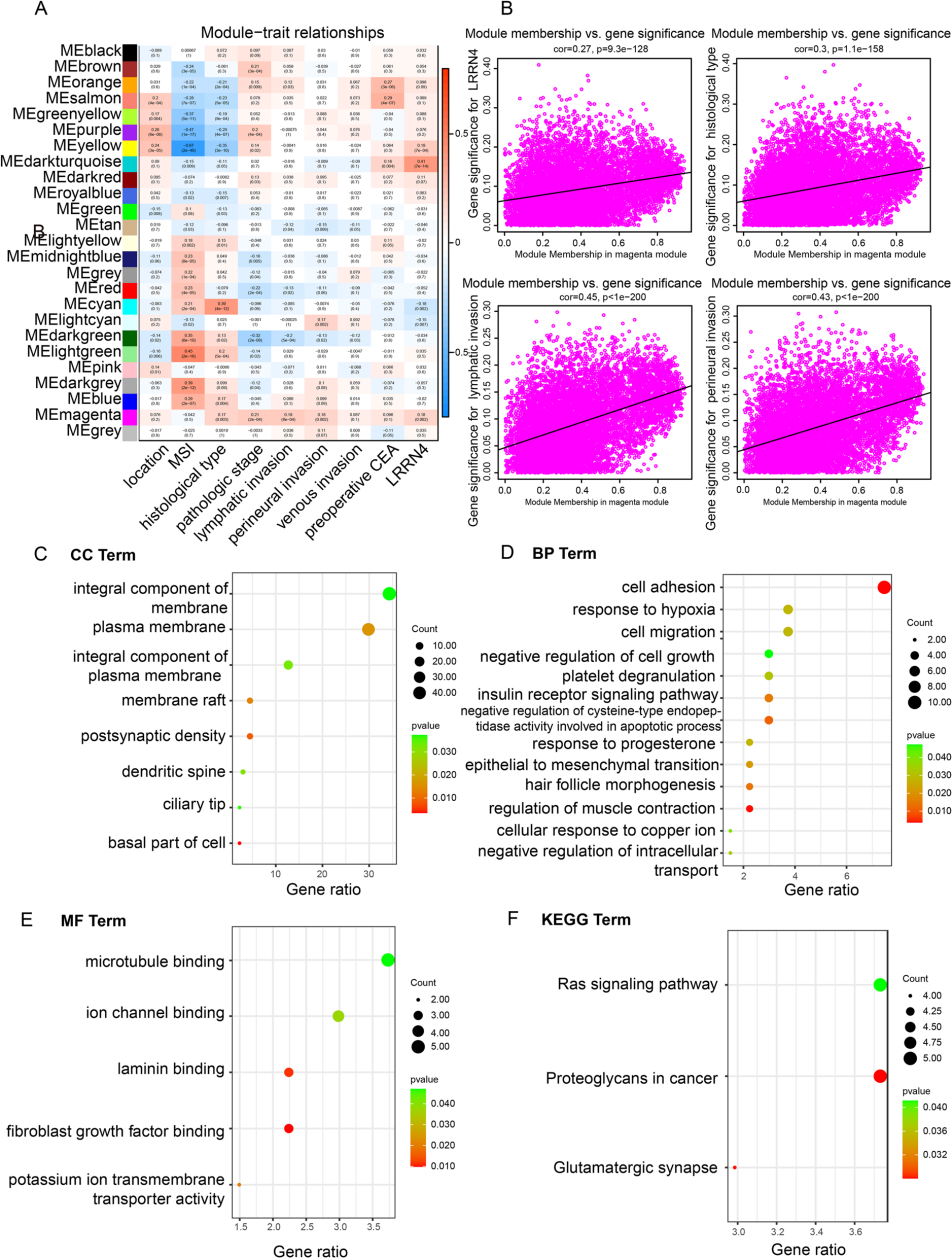
The GO and KEGG pathways of LRRN4-related genes. **A**, **B** Association between gene expression modules and clinicopathologic characteristics and LRRN4 expression. Pearson’s correlation analysis was performed to obtain Pearson’s correlation (p value). **C** The cellular component (CC), **D** biological process (BP), **E** molecular function (MF), and **F** KEGG pathways of LRRN4-related genes in the magenta module, where *p* < 0.05 are displayed

To investigate the potential molecular mechanisms of LRRN4 in CRC, we first used WGCNA to identify gene coexpression modules and link them to LRRN4 expression. We found significant correlations between module eigengenes and the following traits: LRRN4 expression, histological type, lymphatic invasion and perineural invasion. The magenta module was positively associated with LRRN4 expression and several clinicopathological phenotypes, which was further analyzed (Fig. 7A). Next, we calculated the relationship between module membership of the magenta module and gene significance. For most traits, strong correlations were observed (LRRN4: cor = 0.27, *p* = 9.3e−128; histological type: cor = 0.3, *p* = 1.1e−158; lymphatic invasion: cor = 0.45, *p* < 1e−200; perineural invasion: cor = 0.43, *p* < 1e−200), indicating that the genes most representative of the magenta module’s overall expression profile were those most strongly related to LRRN4 expression and clinicopathological traits (Fig. 7B).

To attribute biological meaning to the magenta module, we enriched module significance for the gene ontology and pathway annotations returned from the DAVID database. In total, 8 cell components, 13 biological processes, and 5 molecular functions were enriched (Fig. 7C–E). The enriched biological processes included cell adhesion, response to hypoxia, cell migration and negative regulation of cysteine-type endopeptidase activity involved in apoptotic process, which was linked to malignant features of CRC cells. Microtubule binding, ion channel binding, laminin binding, fibroblast growth factor binding and potassium ion transmembrane transporter activity were highly enriched molecular functions. Meanwhile, the LRRN4-related genes in the magenta module were enriched in several cancer-related pathways, including the Ras signaling pathway, proteoglycans in cancer and glutamatergic synapses, which are reported to be implicated in tumorigenesis and tumor progression, including CRC (Fig. 7F).

**Fig. 8:**
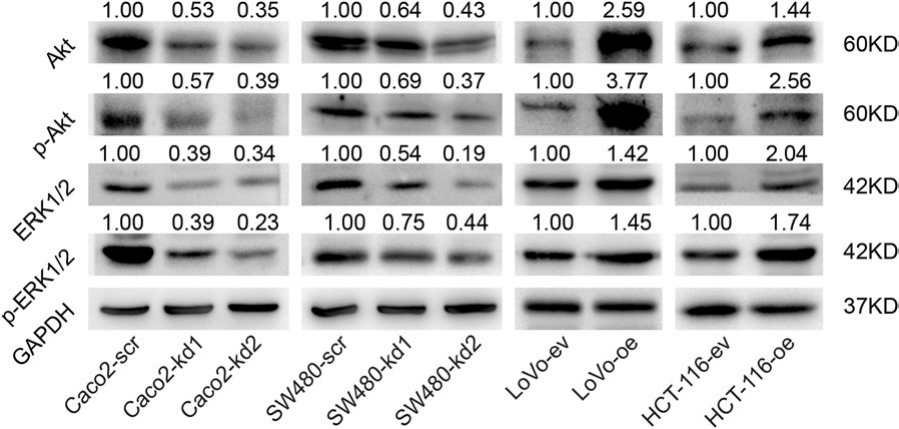
Representative images of western blot analyses of Akt, p-Akt, ERK1/2 and p-ERK1/2 in CRC cells with LRRN knockdown or overexpression

To further verify the reliability of the results obtained by WGCNA combined with DAVID and explore the potential mechanism by which LRRN4 regulated, we measured the expression of Akt, p-Akt, ERK 1/2 and p-ERK1/2, the downstream of the Ras signaling pathway, one of the most important mechanism of CRC development. As Fig. 8 shown, the RAS/MAPK pathway was inhibited after LRRN4 knockdown, and the results of LRRN4 overexpression were opposite of those seen in LRRN4 knockdown cells.

## Discussion

Increasing evidence has shown that the expression of neurogenic biomarkers in cancer cells plays a functional role in crosstalk between cancer cells and neuronal cells to promote carcinogenesis and progression [4–7]. In this study, we first revealed the correlation of LRRN4 expression, a novel neurogenic marker, with CRC at both the mRNA and protein levels. The expression of LRRN4 was high in CRC and was correlated with pathological stage and OS status in the TCGA cohort and our cohort. However, a previous study found that LRRN4 was of low abundance in normal colon tissue and that the level of LRRN4 was much lower in CRC tissue, which was detected by coupling hydroxyapatite chromatography and SDS-PAGE followed by mass spectrometry analysis [18]. In fact, in a few cases, we also found that the expression of LRRN4 is higher in normal tissues than in tumors. This heterogeneous result may be caused by variations in method or sample size. In addition, a high expression level of LRRN4 was associated with poor OS in the TCGA cohort and our cohort. High expression of LRRN4 was statistically associated with poor PFS in the TCGA cohort, while only a trend was found in our cohort, which might be caused by the limited number of patients who developed progression in our cohort. Although the clinical significance of LRRN4 expression in CRC has never been explored before this study, a similar prognostic significance of other members of the LRRN protein family was found in gastric cancer patients [21]. Evidence has shown that members of the LRRN protein family are specifically expressed in nerve tissues and are mainly involved in neuronal development and regeneration [13–15, 21–23]. However, their abnormal expression plays an important role in neurological and nonneurological malignancies, such as neuroblastoma and gastric cancer [21, 24]. Overall, our results suggested the potential functional role of LRRN4 in the progression of CRC and that LRRN4 has prognostic value, similar to other members of the LRRN family.

Although a few reports have demonstrated the differential expression of LRRN4 in primary mesothelioma and CRC patients, the functions of LRRN4 in malignant cells are unclear [17]. To elucidate the role of LRRN4 in the tumorigenesis and progression of CRC, we knocked down or overexpressed LRRN4 in CRC cells. Our results showed that LRRN4 plays a functional role in promoting cell proliferation and DNA synthesis and in inhibiting apoptosis, indicating a function in cancer cells similar to other members of the LRRN family. LRRN1 is involved in the regulation of proliferation and can protect cells from FBS deprivation-induced apoptosis in neuroblastoma cells [25]. Meanwhile, LRRN1 suppresses the apoptosis of gastric cancer cells [21]. Moreover, based on the structural features of LRRN4, it contains not only leucine-rich repeat domains but also a fibronectin type III repeat domain, which has been recognized as a cell adhesion molecule in cell migration [12, 21, 26]. The present results further confirm the promoting role of LRRN4 in cell migration and provide experimental evidence to explain the malignant biological behavior that we observed in CRC patients.

Our results in both CRC patients and cells indicated that LRRN4 plays an oncogenic role in CRC. We further analyzed the potential mechanism of LRRN4 in the CRC malignant phenotype by using WGCNA and DAVID. Several signaling pathways were found to be regulated by LRRN4, including the Ras signaling pathway and the mitogen-activated protein kinase (MAPK) and phosphoinositide-3 kinase (PI3K) pathways, which are known to correlate with the malignant phenotype of cancer cells [27–31]. Our results showed LRRN4 activated RAS/MAPK pathway, knocking down LRRN4 inhibited the expression of p-Akt, p-ERK1/2, while overexpression of LRRRN4 upregulated the expression of p-Akt and p-ERK1/2. These results validated the reliability of the results obtained by WGCNA combined with DAVID, at the same time. There was no evidence suggesting that LRRN directly regulates these signaling pathways before, but LRRN3, a LRRN family member, potentiates Ras/MAPK signaling by facilitating internalization of EGF in clathrin-coated vesicles [23], indicating the closely association between LRRN family and RAS/MAPK pathway. Interestingly, crosstalk among these LRRN4-related signaling pathways (Ras, MAPK, and PI3K pathways) have been reported in cancer. These signaling pathways correlated with the malignant phenotype of cancer cells, including proliferation, cell cycle, apoptosis, epithelial-mesenchymal transition and et al. [30, 32, 33], indicating a crucial regulatory role of LRRN4 in carcinogenesis and progression, as our results indicated.

## Conclusion

In conclusion, our findings have first revealed that LRRN4 is intricately associated with the survival of patients with CRC and is an independent prognostic factor for PFS and OS. Further experiments demonstrated that LRRN4 promotes cell proliferation, DNA synthesis, and migration and suppresses apoptosis in CRC cells, which is mainly due to its activation of RAS/MAPK signaling pathway. Our study highlights the potential of an oncogenic feature of LRRN4, which could be used as a promising prognostic and therapeutic target for CRC.

## Supplementary Information

**Additional file 1:** Expression of LRRN4 in CRC tissue with different clinicopathological characteristics from the TCGA-COREAD cohort.

**Additional file 2:** Kaplan–Meier curves of PFS of CRC in a Chinese cohort based on the expression of LRRN4 protein.

**Additional file 3:** mRNA expression of LRRN4 in different cell lines, and mRNA LRRN4 expression in Caco2 and SW480 cells with LRRN4 knockdown and in HCT-116 and LoVo cells overexpressing LRRN4.

**Additional file 4:** Quantitative analyses of the proportion of the G0/G1, S and G2/M phases in Caco2 and SW480 cells with LRRN4 knockdown and in HCT-116 and LoVo cells overexpressing LRRN4.

**Additional file 5:** Representative images of western blot analyses of cleaved caspase 3 (c-caspase3) in CRC cells knockdown or overexpressing LRRN4 with staurosporine as a positive control.

## Data Availability

The datasets used and/or analyzed during the current study are available from the corresponding author upon reasonable request.

## Abbreviations

CRC: Colorectal cancer
LRRN4: Leucine-rich repeat neuronal 4
DAVID: Database for Annotation, Visualization and Integrated Discovery
WGCNA: Weighted coexpression network analysis
OS: Overall survival
PFS: Progression-free survival
MSI: Microsatellite instability
shRNA: Small hairpin RNA
EdU: 5-Ethynyl-20-deoxyuridine
DMEM: Dulbecco's modified Eagle's medium
SCID: Severe combined immune deficiency
MAPK: Mitogen-activated protein kinases
PI3K: Phosphoinositide-3 kinase
