# Supplementary figures and images for: Prognostic value and biological function of LRRN4 in colorectal cancer
Source: Cancer Cell Int. 2022 Apr 19;22:158. doi: 10.1186/s12935-022-02579-x (PMC9020117; doi:10.1186/s12935-022-02579-x)

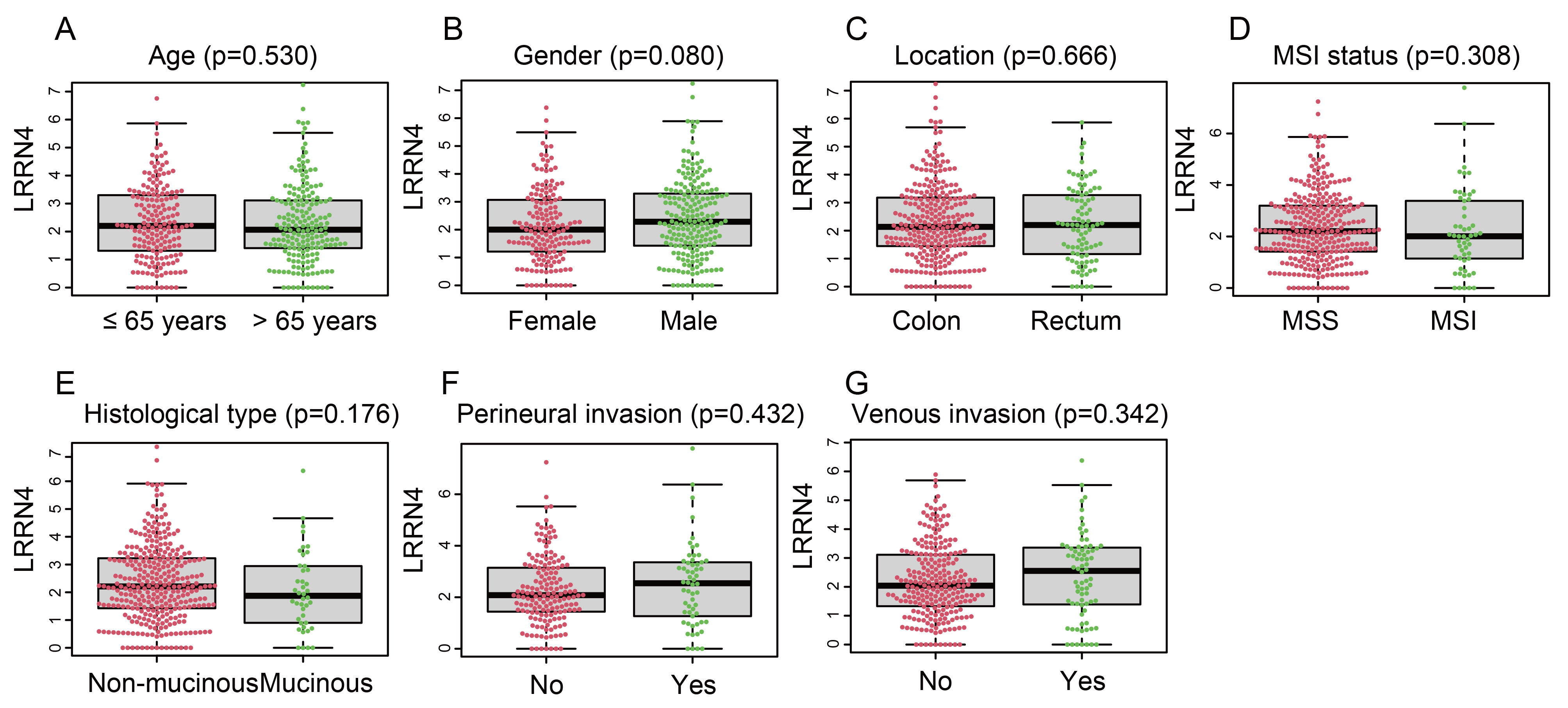

Supplement: Supplementary file 1 — Additional file 1: Expression of LRRN4 in CRC tissue with different clinicopathological characteristics from the TCGA-COREAD cohort. [file 12935_2022_2579_MOESM1_ESM.tif]

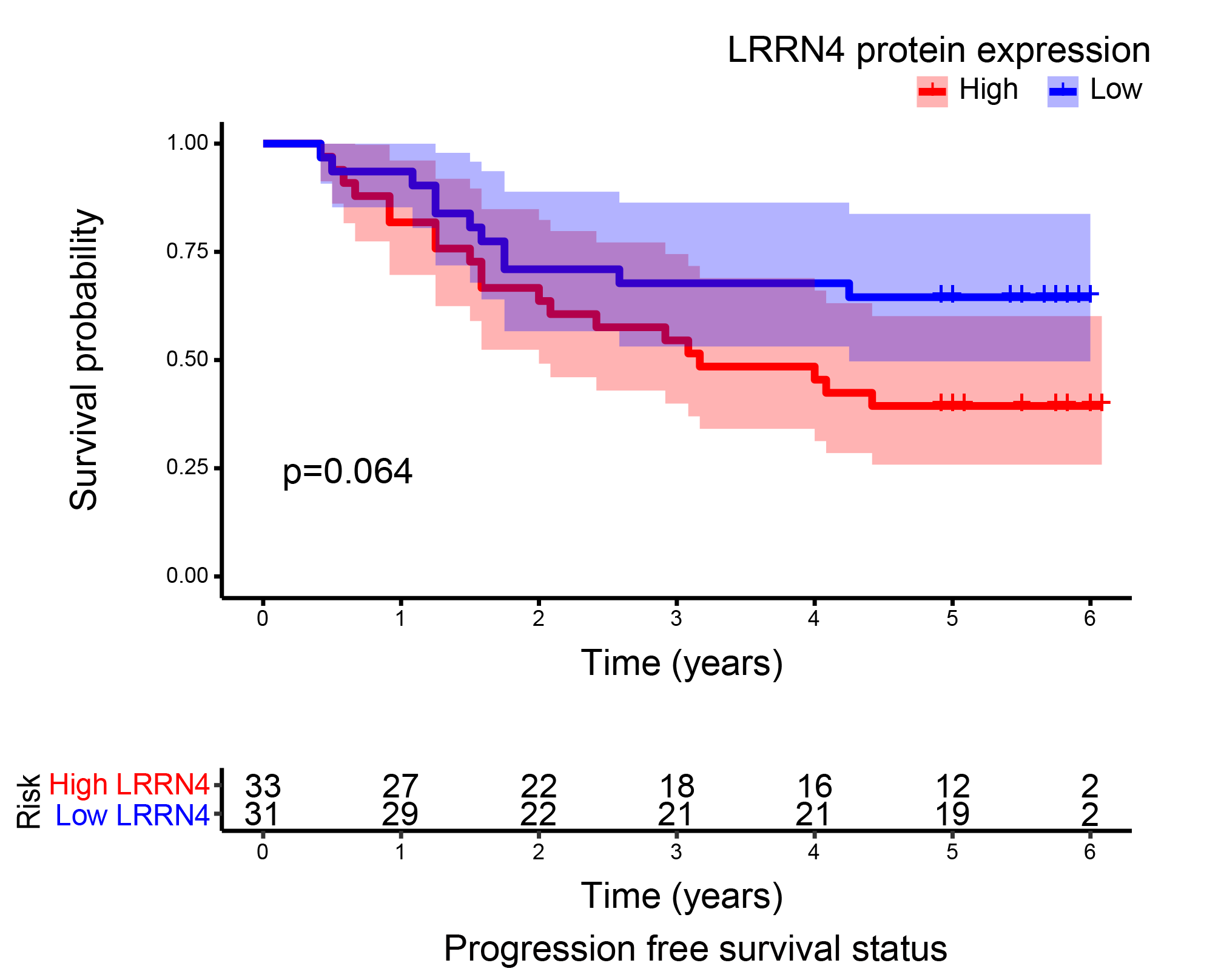

Supplement: Supplementary file 2 — Additional file 2: Kaplan–Meier curves of PFS of CRC in a Chinese cohort based on the expression of LRRN4 protein. [file 12935_2022_2579_MOESM2_ESM.tif]

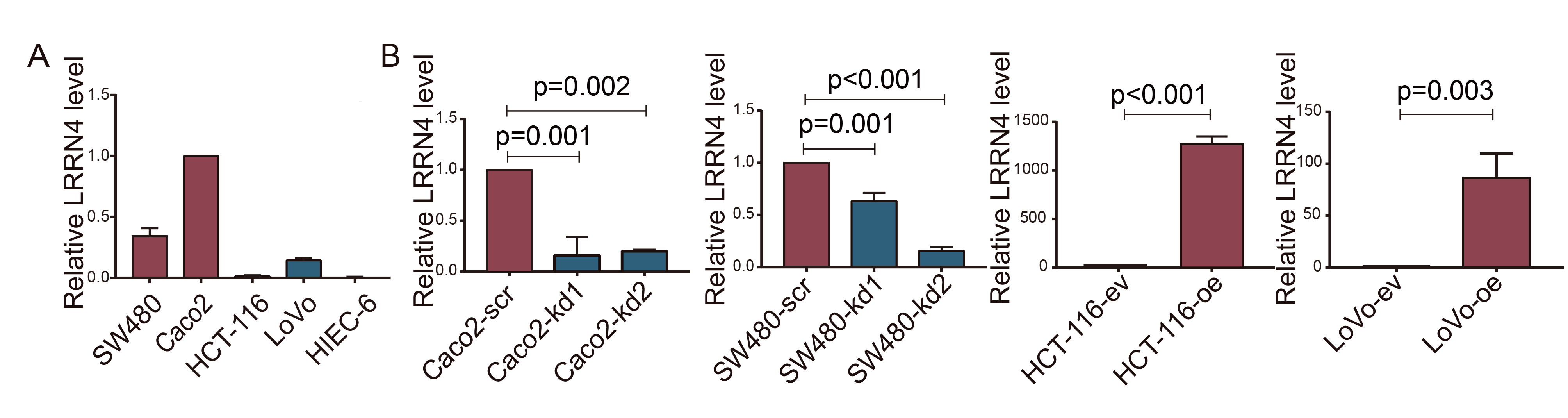

Supplement: Supplementary file 3 — Additional file 3: mRNA expression of LRRN4 in different cell lines, and mRNA LRRN4 expression in Caco2 and SW480 cells with LRRN4 knockdown and in HCT-116 and LoVo cells overexpressing LRRN4. [file 12935_2022_2579_MOESM3_ESM.tif]

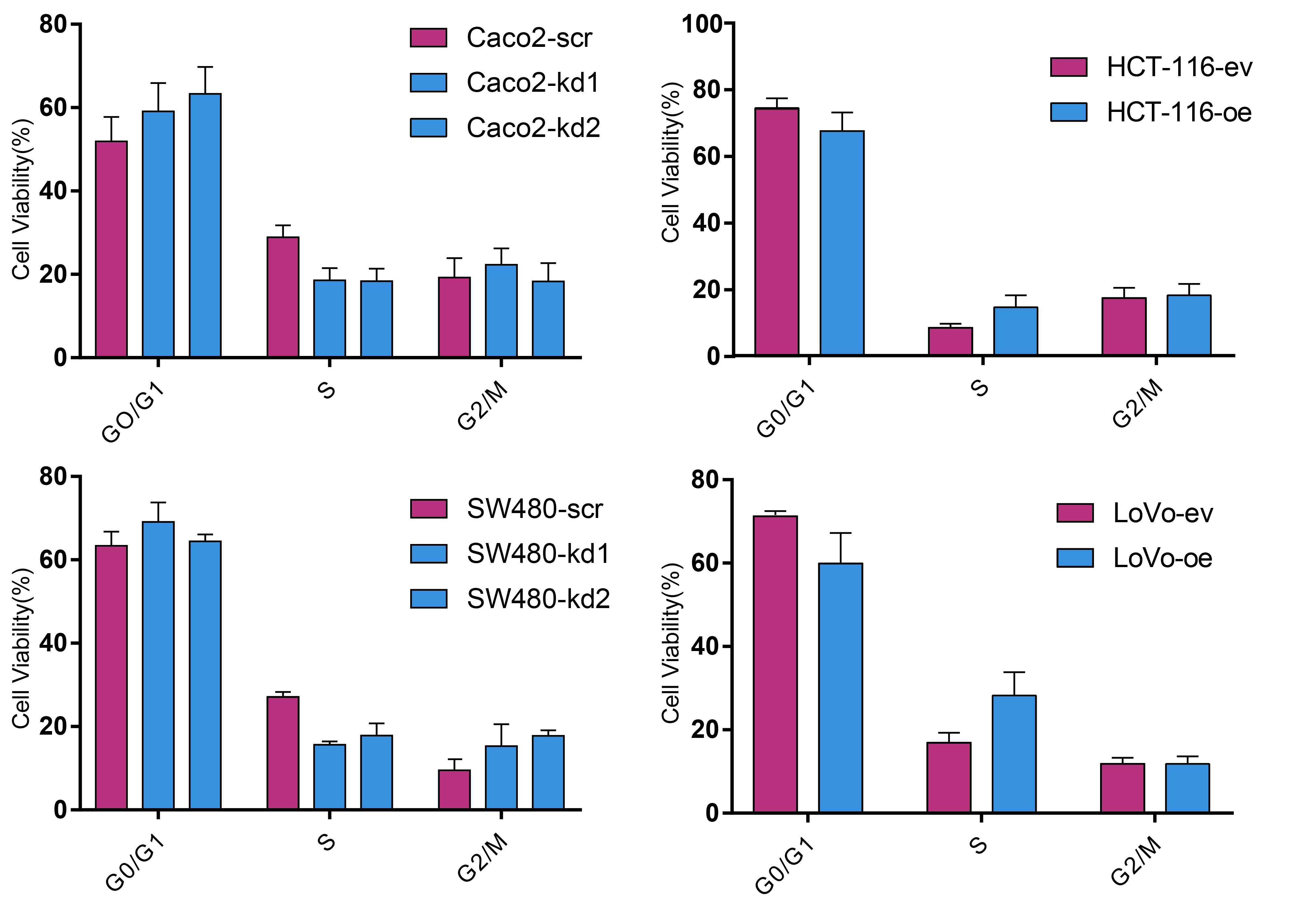

Supplement: Supplementary file 4 — Additional file 4: Quantitative analyses of the proportion of the G0/G1, S and G2/M phases in Caco2 and SW480 cells with LRRN4 knockdown and in HCT-116 and LoVo cells overexpressing LRRN4. [file 12935_2022_2579_MOESM4_ESM.tif]

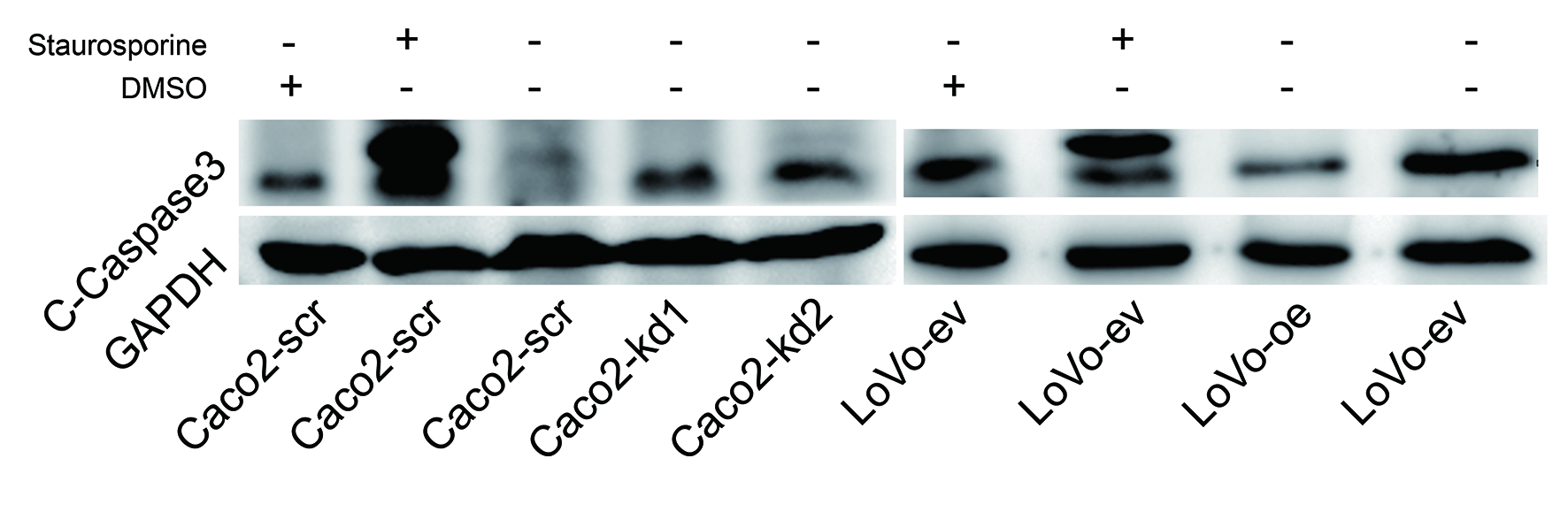

Supplement: Supplementary file 5 — Additional file 5: Representative images of western blot analyses of cleaved caspase 3 (c-caspase3) in CRC cells knockdown or overexpressing LRRN4 with staurosporine as a positive control. [file 12935_2022_2579_MOESM5_ESM.tif]
